# Precision Subtypes of T Cell-Mediated Rejection Identified by Molecular Profiles

**DOI:** 10.3389/fimmu.2015.00536

**Published:** 2015-11-06

**Authors:** Paul Ostrom Kadota, Zahraa Hajjiri, Patricia W. Finn, David L. Perkins

**Affiliations:** ^1^Finn-Perkins Laboratory, Department of Medicine, University of Illinois-Chicago, Chicago, IL, USA; ^2^Finn-Perkins Laboratory, Department of Internal Medicine, Division of Nephrology, University of Illinois-Chicago, Chicago, IL, USA; ^3^Department of Medicine, University of Illinois-Chicago, Chicago, IL, USA; ^4^Department of Surgery, University of Illinois-Chicago, Chicago, IL, USA

**Keywords:** TCMR, precision medicine, personalized medicine, kidney transplant, systems biology

## Abstract

Among kidney transplant recipients, the treatment of choice for acute T cell-mediated rejection (TCMR) with pulse steroids or antibody protocols has variable outcomes. Some rejection episodes are resistant to an initial steroid pulse, but respond to subsequent antibody protocols. The biological mechanisms causing the different therapeutic responses are not currently understood. Histological examination of the renal allograft is considered the gold standard in the diagnosis of acute rejection. The Banff Classification System was established to standardize the histopathological diagnosis and to direct therapy. Although widely used, it shows variability among pathologists and lacks criteria to guide precision individualized therapy. The analysis of the transcriptome in allograft biopsies, which we analyzed in this study, provides a strategy to develop molecular diagnoses that would have increased diagnostic precision and assist the development of individualized treatment. Our hypothesis is that the histological classification of TCMR contains multiple subtypes of rejection. Using R language algorithms to determine statistical significance, multidimensional scaling, and hierarchical, we analyzed differential gene expression based on microarray data from biopsies classified as TCMR. Next, we identified KEGG functions, protein–protein interaction networks, gene regulatory networks, and predicted therapeutic targets using the integrated database ConsesnsusPathDB (CPDB). Based on our analysis, two distinct clusters of biopsies termed TCMR01 and TCMR02 were identified. Despite having the same Banff classification, we identified 1933 differentially expressed genes between the two clusters. These genes were further divided into three major groups: a core group contained within both the TCMR01 and TCMR02 subtypes, as well as genes unique to TCMR01 or TCMR02. The subtypes of TCMR utilized different biological pathways, different regulatory networks and were predicted to respond to different therapeutic agents. Our results suggest approaches to identify more precise molecular diagnoses of TCMR, which could form the basis for personalized treatments.

## Introduction

An important goal in medicine is to develop precision therapies specific to each individual to deliver personalized medicine. As eloquently stated by Sir William Osler over 100 years ago, “variability is the law of life, and as no two faces are the same, so no two bodies are alike, and no two individuals react alike and behave alike under the abnormal conditions we know as disease.” Since the introduction of cyclosporine in the late 1980s, the therapeutic protocols for many patients in clinical transplantation have been based on three types of immunosuppressive drugs: a calcineurin inhibitor, an antimetabolite, and a steroid. More recently, an array of new agents including biological agents have emerged or are entering investigational study. In addition, protocols that are calcineurin inhibitor free or steroid sparing have also been developed. Given the increasing number of therapeutic agents and potential protocols and the limited number of transplant patients, it is not tractable to evaluate all of the potential therapeutic permutations in prospective clinical trials. Furthermore, a strategy is needed to precisely identify the optimal therapy to apply personalized medicine for each individual patient based on each patient’s genotype and phenotype (1–7).

Since its development in 1993, the Banff Classification has served as a valuable tool in the diagnosis of allograft rejection and a guide for clinical management. The first Banff classification standardized the histopathological criteria to diagnose rejection into six major categories based on the histopathological findings (8): (1) normal, (2) hyperacute, (3) borderline mild tubulitis, (4) acute rejection, (5) chronic allograft nephropathy (CAN), (6) other changes not due to rejection. A numerical grading was introduced for each of the renal compartments. This classification created a framework for further changes and modifications in subsequent updates of the Banff classification. Despite the success and huge positive impact this classification had on transplant medicine, it also had limitations. The major weaknesses are the substantial variability among pathologists and the lack of an external validation tool (9). The reproducibility of the Banff classification was assessed with a Kappa statistics scoring system (10–12). These studies showed a moderate reproducibility score for diagnostic classification, whereas the numerical grading of tubulitis had an extremely low kappa score indicating low reproducibility (10). Identifying a correlation between the Banff classification and graft survival has also been a challenge. In 2015, Krisl et al. followed 182 patients who developed a rejection episode for a median of 527 days. They noted no difference in death censored graft survival in the first 6 months after transplantation between the acute cellular rejection grades IA, IB, IIA, and IIB. They also noted no difference between the early and late Banff IA or IB classifications. However, the same histological classification of IIA had a significant difference in graft survival if it occurred late (after 6 months) versus early (13). These differences suggest that the different subtypes do not represent a graded severity score that correlates with graft survival, but rather a different type of rejection. In another study, Wu et al. followed 270 patients with rejection and noted no significant difference in the graft survival between TCMR I, II, or III. However, they noted worse graft outcomes in patients with vascular involvement regardless of the degree of the timing or the degree of interstitial or tubular involvement (14). Again, this analysis suggests different subtypes of TCMR that are not precisely captured by the Banff classifications. In summary, these studies demonstrate the limitations of the Banff classification in grading the severity of rejection and in predicting outcomes.

Our overall hypothesis is that the histological diagnosis of TCMR as defined by the Banff classification of kidney transplant biopsies contains multiple subtypes of rejection involving different biological pathways and functions. To address these goals, in this report, we use a systems biology approach to provide a proof of principle analysis that identifies potential therapeutic agents that target specific subtypes of T cell-mediated rejection (TCMR).

The basis of our approach is the analysis of the transcriptome in allograft biopsies. The analysis of differential expression of mRNA has several advantages. First, genome wide assays of the transcriptome are relatively quick, quantitative, and reproducible. In contrast, other “omic” technologies, in particular proteomics and metabolomics can be technically more challenging and not genome wide. Second, the level of gene expression quantitated in the transcriptome reflects multiple effects including genomic, epigenetic, metagenomic, and environmental influences and thus integrates the effect of multiple biological regulatory mechanisms. Based on these considerations, analysis of the transcriptome, which is utilized in this study, is a quantitative, reproducible, and cost-effective approach to assay a genome wide response.

To interpret genome wide data, the application of systems biology methods that analyze pathways and networks of molecules in an “interactome” increases confidence in functional biological interpretations compared with the reductionist approach of analysis of isolated molecular interactions. In our analysis, we first identified the significant changes in the transcriptome of microarray data in a public database of kidney transplant biopsies that were classified as TCMR or control. After excluding outliers using multidimensional scaling (MDS), which is an essential step that supports precision analysis, we identified subtypes of TCMR using unsupervised hierarchical clustering. For each subtype, we constructed a protein–protein interaction (PPI) network, a gene regulatory network, and a KEGG pathway analysis, which illuminated interaction networks, signaling pathways, and regulatory mechanisms. In addition, we analyzed the DrugBank database of candidate drugs to identify putative therapeutic agents that would be specific for each subtype of TCMR.

## Materials and Methods

### Data

The data files were downloaded from NCBI through R, version 3.10, using a *Bioconductor* package, GEOquery (15, 16). The dataset contains the microarray expression from an HG-U133_Plus_2 Affymetrix Human Genome array. Gene expression was given as log2 fold change against controls. The dataset included 202 kidney biopsies taken from renal transplant patients undergoing biopsies for cause (17). The expression and phenotypic data can be found on the Gene Expression Omnibus (GEO) database, using GEO ascension number (*GSE21374*) (18).

The initial data set of 202 kidney biopsies included eleven diagnoses based upon pathology report. These biopsies included rejection diagnoses of antibody-mediated rejection (ABMR), acute tubular necrosis (ATN), BK virus, borderline, CAN, calcineurin inhibitor toxicity (CNIT), glomerulonephritis (GN), tubular atrophy/interstitial fibrosis (TAIF), T cell-mediated rejection (TCMR), and transplant glomerulopathy (TGP). There were 143 samples taken from 85 patients that underwent renal transplant. Eight additional kidney biopsies were taken from normal, native kidneys from patients undergoing nephrectomy for renal cell carcinoma. Finally, 51 additional transplant biopsies were added as a validation set. We focused on the largest cohort, TCMR, using the renal nephrectomy samples as a baseline control.

### Statistical Methods

An analysis pipeline was created in the R language statistical and graphing environment (19). First, we normalized the 54,675 Affymetrix probe sets by *Z*-score and filtered the data based on the scaled expression level >0.12 and coefficient of variation (CV), which selected 6,473 genes that showed high expression and high CV. Next, we identified outlier samples using MDS. Samples with an intracentroid distance >2 SD greater than the mean were classified as outliers and removed from further analysis. To identify subtypes of TCMR, we performed unsupervised hierarchical clustering using the stats package. The clusters were evaluated by connectivity, Dunn, and Silhouette index, which identified two subtypes of TCMR. Differential gene expression was determined by student’s *t* test (*p* < 0.05). Correction for multiple testing was performed by false discovery rate algorithm (fdr <0.05).

### Molecular Interaction Analysis

We used the ConsesnsusPathDB (CPDB), hosted by the Max Plank Institute of Molecular Genetics (20). CPDB combines 32 public resources for biological interactions including the KEGG, BIND, DrugBank and MINT databases. For each database, we analyzed TCMR01, TCMR02, and core gene list independently.

Over-representation analysis was performed in CPDB for KEGG pathways. The resulting enriched pathway-based sets included a minimum of two input genes with a hypergeometric test *p*-value of 0.01. Edges between KEGG pathways included at least a 30% overlap between connecting nodes.

We used CPDB induced network module analysis of high-confidence PPI, gene regulatory interactions, and drug-target interactions.

### Network Visualization

Network data were visualized with the imaging platform Cytoscape (21).

## Results

### Defining Subtypes of T Cell-Mediated Rejection

**Figure 1:**
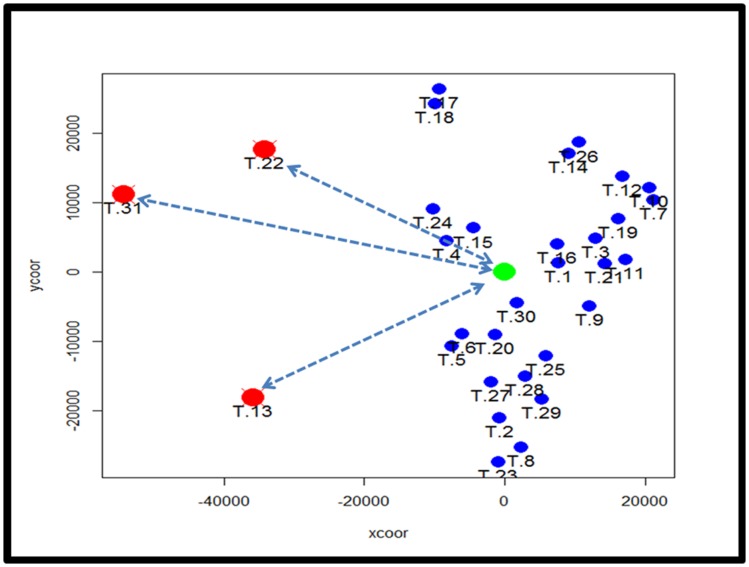
**Analysis of potential sample outliers with multidimensional scaling (MDS)**. We analyzed 31 samples with a pathological diagnosis of T cell-mediated rejection (TCMR) in the kidney transplant database (GSE21374). Using multidimensional scaling of the gene expression data, we identified samples with an intracentroid distance *z*-score >2 (represented by the dashed lines) which included three samples (T13, T22 and T31).

**Figure 2:**
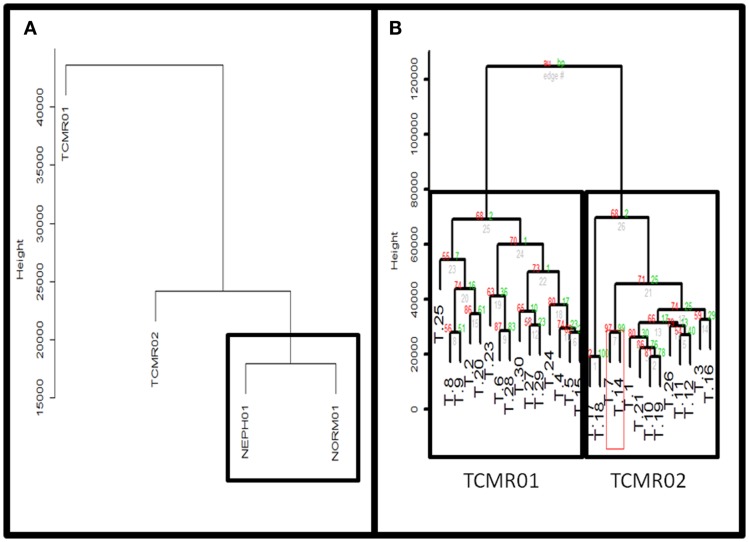
**(A)** Comparison of TCMR01 and TCMR02 with the normal and nephrectomy controls. Unsupervised hierarchal clustering of TCMR01, TCMR02, NEPH01 (nephrectomy) and NORM01 (normal biopsy samples). **(B)** The subtypes of T cell-mediated rejection (TCMR) classified by unsupervised hierarchical clustering. After filtering the initial 54,675 Affymetrix probe sets based upon scaled expression and coefficient of variation, we identified two distinct groups of T cell-Mediated Rejection: TCMR01 (left) and TCMR02 (right). The two sub-diagnoses were differentiated by 1933 significant genes based upon false-discovery rate corrected *p*-values of 0.05.

To identify subtypes of TCMR rejection in kidney allografts, we analyzed all samples classified as TCMR rejection (and nephrectomy controls) using the Banff histological classification of rejection in a database of kidney transplant biopsies (GSE 21374). First, we filtered microarray expression data based on the CV >0.12. Next, we analyzed the 31 samples classified as TCMR using MDS to identify statistical outliers among the samples (Figure 1). We defined samples as outliers that were more than two SD from the medoid. Based on this criterion, we classified three samples (T.31, T.22, and T.13) as outliers. The remaining samples were analyzed by hierarchical clustering using stats Package in R language (Figure 2B). To determine the optimal number of clusters, we analyzed the dendrogram based on connectivity, Dunn Index, and Silhouette Index (Table S1 in Supplementary Material). All three methods supported partitioning the results into two distinct clusters that we termed “TCMR01” and “TCMR02.”

### Hierarchical Clustering Based on Molecular Heterogeneity

Next, we calculated the mean expression levels of genes in TCMR01, TCMR02, NEPH01 (nephrectomy control), and NORM01 (biopsies pathologically classified as normal) and analyzed the subtypes with hierarchical clustering (Figure 2A). NEPH01 and NORM01 were similar based on proximity in the dendogram. Interestingly, TCMR01 was more dissimilar than TCMR02 from the NEPH01 and NORM01 subtypes suggesting that TCMR01 exhibited the more extreme subtype. To determine if the two clusters contained differentially expressed genes, we performed *t*-tests between TCMR01 and TCMR02, which identified 1933 genes that were significantly differentially expressed (fdr <0.05) (Table S2 in Supplementary Material). Thus, although the TCMR01 and TCMR02 subtypes of rejection had similar histological diagnoses, they were markedly heterogeneous at the molecular level of gene expression.

### Functional Differences Between Subtypes of T Cell-Mediated Rejection

**Figure 3:**
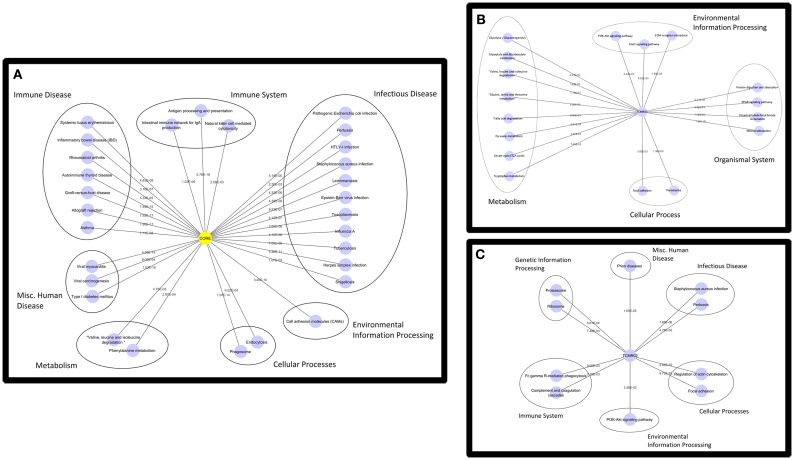
**Functional analysis of the KEGG pathways in the core (A), TCMR01 (B), and TCMR02 (C) subtypes**. We identified KEGG pathways for the significantly modulated genes in the three subtype. The five main functional pathways are presented in the figures. Allograft rejection pathways along with other immune pathways are highly expressed in the core group, whereas the TCMR01 is rich with metabolic pathways. Complement and coagulation cascades seem to be uniquely expressed in the TCMR02 group. The numbers on the lines represent the *p*-values and are all significant with a value of <0.05. The data was analyzed with CDBP and the networks were visualized with Cytoscape.

Based on the large number of differentially expressed genes in the TCMR01 and TCMR02 subtypes, we investigated whether the two subtypes had different biological functions. We selected three groups of differentially expressed genes: a core group that was differentially expressed in both subtypes and groups that were uniquely differentially expressed in either TCMR01 or TCMR02. Next, we identified the KEGG pathways that were significantly associated with each group of genes (Figure 3; Table S3 in Supplementary Material). In the core group, defined as the genes common to both TCMR01 and TCMR02, the pathway with the highest significance was “allograft rejection” (*q* < 9.67E−16), which supported the validity of our approach. Additional highly significant pathways included “graft-versus-host disease” (*q* < 2.10E−15), “antigen processing and presentation” (*q* < 2.10E−15), “type I diabetes mellitus” (*q* < 2.90E−15), “autoimmune thyroid disease” (*q* < 4.75E−14), “viral myocarditis” (*q* < 9.44E−14), “phagosome” (*q* < 1.27E−10), and “cell adhesion molecules” (*q* < 3.37E−09). All of the significant pathways share a strong pathological immune response and the emergence of pathways associated with autoimmunity, allergy and infections in addition to alloimmunity are due to the overlap in the genes involved in these immune processes. Importantly, it is notable that the significance level of “allograft rejection” is more than an order of magnitude more significant than the other pathways. In addition to immune processes, we also detected significant metabolic pathways including “tryptophan metabolism” (*q* < 0.000152), “histidine metabolism” (*q* < 0.000194), “glycine, serine, and threonine metabolism” (*q* < 0.00154), “fatty acid degradation” (*q* < 0.00242), “valine, leucine, and isoleucine degradation” (*q* < 0.00285), “arginine and proline metabolism” (*q* < 0.0074), and “fructose and mannose metabolism” (*q* < 0.00864) that may be important in modulating the immune response and could potentially serve as novel therapeutic targets. In contrast to the core response, the pathways associated with the TCMR01 subtype predominantly involved metabolism. The pathways represented a diverse array of metabolic functions ranging from the citrate cycle to amino acid metabolism to glyoxylate metabolism to extracellular matrix interaction. The TCMR01 subtype did not contain any significant pathways directly involving immune functions, whereas the TCMR02 included a number of immune responses associated with infections, complement and coagulation cascades and natural killer cell-mediated cytotoxicity. Based on the KEGG pathway analysis, our data indicate that the TCMR01 and TCMR02 subtypes of rejection, in addition to differential gene expression, have different biological functions.

### Protein Interactions

**Figure 4:**
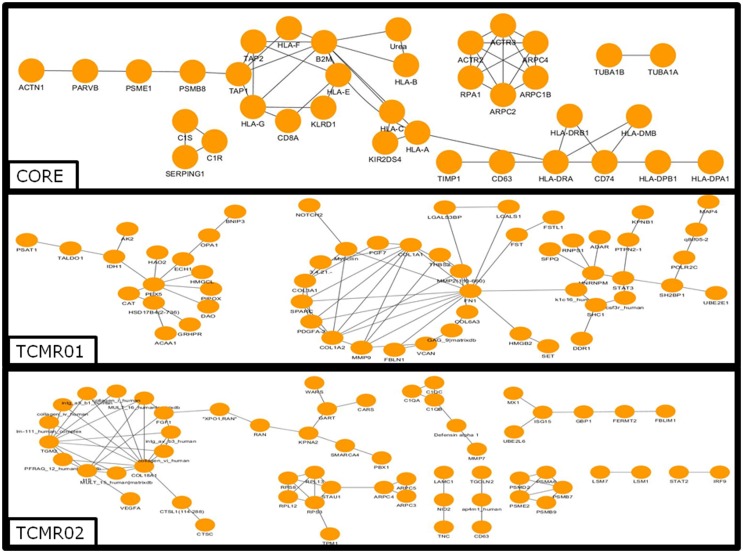
**Analysis of the protein–protein interaction networks in the core, TCMR01 and TCMR02 subtypes**. We identified the protein–protein interaction networks of the significantly modulated genes for the core, TCMR01 and TCMR02. Each node represents a protein from the input gene list, and each edge shows a high-confidence protein–protein interaction (PPI). The three subnetworks show how the TCMR subtypes are vastly different on a molecular level. The data was analyzed with CDBP and the networks were visualized with Cytoscape.

To investigate the mechanisms regulating the different biological functions in the different subtypes of rejection, we constructed PIP graphs (Figure 4). Overall, it is apparent that the PIPs include subnetworks involved a diverse array of biological processes. For example, the core genes common to both subtypes mediated upregulation of HLA class I and II molecules, expression of cytoskeletal molecules including tubulin, fibulin, and actin-binding proteins, proteasome components, IFNγ receptor, and interferon response factors and regulators of stress and energy production. The TCMR01 subtype upregulated collagen and metabolic enzymes. In contrast, the TCMR02 subtype had increased expression of RANTES, complement components of C1q, matrix protein keratin 19, cytoskeletal components of actin and tropomyosin, proteasome components, and GTP-binding proteins.

### Transcription Factors

**Figure 5:**
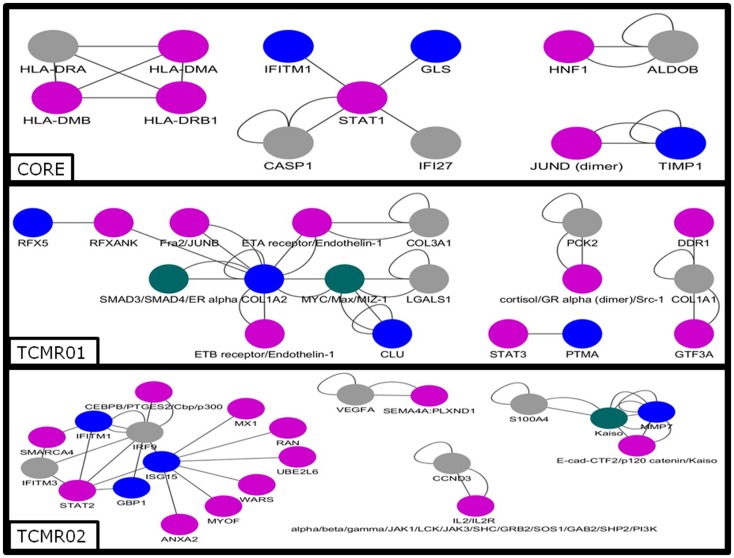
**Analysis of the gene regulatory interaction networks in the core, TCMR01 and TCMR02 subtypes**. We identified putative gene regulatory networks in the core, TCMR01 and TCMR02 based on the significantly modulated genes (blue nodes). The contrasting elements in the two subnetworks show that the TCMR subtypes are different on the level of transcriptional regulation. The data was analyzed with CDBP and the networks. Gray nodes represent products. Purple nodes represent transcription factors. Green nodes represent repressors.

Next, we analyzed the mechanisms regulating the differential gene expression by focusing on the regulation of gene expression by transcription factors (Figure 5). In the core genes, we detected STAT1, JUND, HNF1, and IKB. In contrast, transcription factors unique to the TCMR01 subtype include STAT3, FRA, JUNB, MYC, GR, and ZIC1, whereas transcription factors unique to the TCMR02 subtype include STAT2, MAFb, Kaiso, EGR1, and CEBPd. In addition, FKBP5, which is a *cis–trans* prolyl isomerase that mediates calcineurin inhibition and binds the immunosuppressants, FK506 and rapamycin is upregulated in TCMR02. The differential abundance of transcription factors, which are known to be regulated by different cytokines and growth factors, suggest the activation of different signal transduction pathways in the two rejection subtypes.

### Drug–Target Interactions

**Figure 6:**
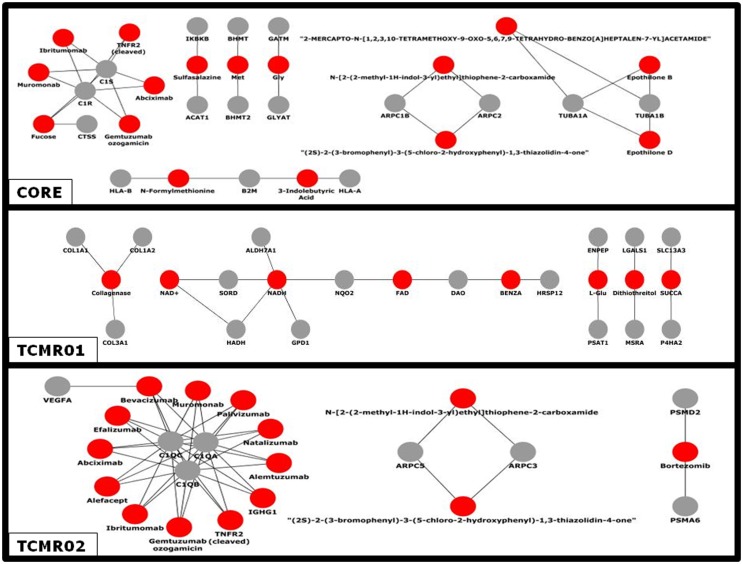
**Analysis of the network of potential drug-targets**. Potential drugs (red nodes) that target proteins (gray nodes) that were significantly modulated in the core, TCMR01 and TCMR02 subtypes were identified. The majority of these drug-target relationships have been curated by the literature associated by DrugBank. The data was analyzed with CDBP and the networks were visualized with Cytoscape.

Given the different transcription factor and effector mechanisms functioning in the different subtypes, we investigated the notion that different treatment modalities would be more precise for each subtype of rejection. An analysis of the potential therapeutic drugs effective against the core genes containing both TCMR01 and TCMR02 phenotypes identifies multiple drugs that have been studied in clinical trials or FDA approved as treatment for transplantation including Muromonab (OKT3), Epothilone B, Epothilone D, Gemtuzumab ozogamicin, Ibritumonab, PDX, Fucose, Sulfasalazine, and TNRF2 (cleaved) (Figure 6). In an analysis of drugs that may be more precisely targeted to each subtype, we identified, as expected based on the PIP, drugs targeted to metabolic processes in TCMR01. In contrast, among the drugs targeted to TCMR02, we detected 15 agents in clinical use or that have undergone clinical trials including Alemtuzumab, Alefacept, and Gemtuzumab. In addition, several potential cancer drugs had potential targets in the TCMR02 subtype. These results suggest that our analytical methods may identify therapeutic drugs that would be more precise in the treatment of subtypes of TCMR.

## Discussion

In this study, we investigated the hypothesis that the histological classification of TCMR contained multiple subtypes of rejection involving different biological pathways and functions. Our analysis using systems biology approaches identified two distinct subtypes of rejection, TCMR01 and TCMR02. We confirmed the validity of the clusters by three criteria (connectivity, Dunn index, and Silhouette index). A direct comparison of gene expression between the two subtypes identified 1933 genes that were significantly differentially expressed (fdr <0.05) despite the fact that all samples had a similar histological diagnosis. We suggest that the significance of our study is the demonstration of the proof of principle that analysis of the transcriptome may be a more precise classifier than current histological diagnoses.

We identified three groups of differentially expressed genes that composed the core response defined as the genes common to both subtypes and groups unique to each subtype of rejection. A functional analysis of gene expression data using the KEGG database identified different functions for each group (18). For example, the core functions that were based on genes differentially expressed in both subtypes included immune functions involved in alloimmune responses. In fact, the most significant pathway was “allograft rejection,” which strongly supports the validity of our analysis. In contrast, the KEGG pathways associated with the unique genes in TCMR01 included predominantly metabolic functions (e.g., glyoxylate metabolism, amino acid metabolism, and citrate cycle), which may indicate parenchymal damage to the graft. The pathways unique in TCMR02 included complement and coagulation cascades and natural killer cell cytotoxicity. Thus, at the functional level our analysis demonstrated core functions common to both subtypes as well as unique functions specific for either TCMR01 or TCMR02. The unique functions suggest potential strategies to develop precision treatments, potentially applicable to transplant rejection.

We analyzed the KEGG database, which is collated on current knowledge (22). It is notable that multiple pathways activated in the core genes were either immune or metabolic processes. For example, another immune pathway was graft-versus-host disease; however, the patients did not have clinical evidence of graft-versus-host disease. Importantly, this pathway was more than one order of magnitude less significant than allograft rejection and a manual inspection of the relevant genes in these pathways shows that the identical genes can participate in multiple broad disease focused KEGG pathways, such as allograft rejection or GVHD. Thus, when evaluating these disease pathways, it is essential to include additional criteria such as clinical correlates or metadata. When considering the more biologically fundamental pathways (e.g., phagosome, histidine metabolism, proteasome, fatty acid degradation, valine degradation, endocytosis, natural killer cell cytotoxicity, arginine metabolism, and fructose metabolism), we observed that the relevant genes are activated.

We also investigated potential mechanisms that mediated the disease processes identified by KEGG pathways. At the level of protein interactions, the core response includes anticipated immune molecules including HLA Class I and II, CD8, and complement receptors. In addition, we identified antigen processing and presenting molecules (HLA-DMβ, proteasome, and CD74) cytoskeletal proteins (tubulin, actin). In contrast, the TCMR01-specific proteins included numerous metabolic mediators and collagen proteins suggesting possible wound healing and fibrosis. The TCMR02-specific proteins included C1q complement proteins, FKBP, RANTES, and cathespin O. At the level of differential expression of transaction factors, there were differences among the three groups. Interestingly, an analysis of the STAT molecules showed that the core response included STAT1, TCMR01 included STAT3, and TCMR02 included STAT2 indicating the activation of different signal transduction pathways in each group.

Considered as a whole, we emphasize that the most compelling observation of our systems biology approach is the demonstration that diverse biological mechanisms are coordinated in an integrated response in allograft rejection. As expected, multiple immune mechanisms are identified (e.g., MHC Class I and II, complement pathways, cytokines, and chemokines). In addition, genes involved in regulation of the cytoskeleton, extracellular matrix, metabolism, gene regulation, apoptosis, signal transduction, and stress response were identified. Importantly, diverse regulatory mechanisms that could potentially coordinate the regulation of these mechanisms were also identified including regulators of transcription, RNA processing, splicing and stability, translation, nuclear transport, chromatin remodeling and epigenetic modifications. The subnetworks and pathways depicted in our analyses identify some of the molecular interactions that may regulate and coordinate the biological systems in TCMR.

Bunnag et al. analyzed the relationship between the molecular expression, histopathology, and renal function in kidney transplant biopsies with low GFR using microarray technology. They analyzed transcripts differentially expressed in patients with low versus high GFR. They noted that the highest expressed were tissue injury transcripts, whereas the lowest expressed were the kidney parenchyma transcripts (23). Genes which were highly expressed in the tissue injury pathway included integrins, keratin genes and a metalloproteinase gene (MMP7) were noted in our TCMR02 group. This indicates that our analysis of rejection subtypes identifies some of the same genes. A major difference between the studies is that the study population analyzed by Bunnag et al. only included 13% biopsy proven TCMR. Einecke et al. investigated whether there is a specific transcriptome indicative of organ failure when the biopsy is performed 1 year after transplantation. A direct analysis between our gene data set and their gene data set shows a 50% shared genes in the core and TCMR01 groups and 37% with the TCMR02 group (Supplementary Material) (17). Their study population had multiple causes of graft failure and only 13% had TCMR. The authors developed a gene expression risk score which was predictive of graft failure in biopsies of kidney transplants greater than 1 year after transplantation. Their analysis differs from ours as they did not focus on TCMR but rather analyzed all biopsies with graft dysfunction. As expected, the genes associated with graft failure had functions related to tissue injury. Sarwal et al. analyzed microarray data from 59 pediatric renal allograft biopsies with poor function. They identified three different signatures of the transcriptome although the samples were indistinguishable by light microscopy (24). Their study re-emphasizes the need to establish a genetic classification which will aid in the management of TCMR, which is the problem our study addressed. We analyzed microarray data of only TCMR biopsies and classified the results into two major subtypes according to their functional pathways. Our detailed approach allowed us to identify specific pathways, which may be targeted in the future with specific therapies for TCMR01 or TCMR02 subtype of rejection. This new classification could provide specific therapeutic targets for personalized treatment.

Our study identified two specific subtypes of rejection, TCMR01 or TCMR02. As expected, we also identified drugs that had putative targets in both subtypes. Interestingly, a number of the drugs that were potential therapies are either current or previous therapies (Muromonab, Alemtuzumab, and Alefacept), are in clinical trials or are treatments for other diseases including cancer (Abciximab, Gemtuzumab ozogamicin, Ibritumonab, sunitinib, Bevacizumab, CPG52364, mc0457, mln518, ptk787, Ranibizumab, PDX, Efalizumab, and Natalizumab) (25–40). The fact that some of these agents have been clinically effective in the treatment of rejection (e.g., Muromonab) supports the validity of the analysis and suggests that it could be informative to develop clinical trials that link therapeutic agents with a specific molecular classification, either TCMR01 or TCMR02. In addition, future studies that combine integrated omics analyses, e.g., combining proteomics, metabolomics, etc., with transcriptomics could increase the power of a systems analysis.

## Author Contributions

DP designed the systems biology approach of the project. PF and DP provided direction for the analyses and interpretation of the data. ZH, PF, and DP provided clinical insights to genetic output. PK implemented the statistical calculations and created the network visualizations.

## Conflict of Interest Statement

The authors declare that the research was conducted in the absence of any commercial or financial relationships that could be construed as a potential conflict of interest.
